# The 15-Months Clinical Experience of SARS-CoV-2: A Literature Review of Therapies and Adjuvants

**DOI:** 10.3390/antiox10060881

**Published:** 2021-05-31

**Authors:** Alessio Danilo Inchingolo, Gianna Dipalma, Angelo Michele Inchingolo, Giuseppina Malcangi, Luigi Santacroce, Maria Teresa D’Oria, Ciro Gargiulo Isacco, Ioana Roxana Bordea, Sebastian Candrea, Antonio Scarano, Benedetta Morandi, Massimo Del Fabbro, Marco Farronato, Gianluca Martino Tartaglia, Mario Giosuè Balzanelli, Andrea Ballini, Ludovica Nucci, Felice Lorusso, Silvio Taschieri, Francesco Inchingolo

**Affiliations:** 1 Department of Interdisciplinary Medicine, University of Medicine Aldo Moro, 70124 Bari, Italy; ad.inchingolo@libero.it (A.D.I.); giannadipalma@tiscali.it (G.D.); angeloinchingolo@gmail.com (A.M.I.); luigi.santacroce@uniba.it (L.S.); mtdoria51@gmail.com (M.T.D.); drciroisacco@gmail.com (C.G.I.); francesco.inchingolo@uniba.it (F.I.); 2 Department of Medical and Biological Sciences, University of Udine, Via delle Scienze, 206, 33100 Udine, Italy; 3 Research at Human Stem Cells Research Center HSC, Ho Chi Minh 70000, Vietnam; 4 Embryology and Regenerative Medicine and Immunology, Pham Chau Trinh University of Medicine Hoi An, Hoi An 70000, Vietnam; 5 Department of Oral Rehabilitation, University of Medicine and Pharmacy “Iuliu Hatieganu”, 400012 Cluj-Napoca, Romania; candreasebastian@yahoo.com; 6 Department of Pedodontics, County Hospital Cluj-Napoca, 400000 Cluj-Napoca, Romania; 7 Department of Innovative Technologies in Medicine and Dentistry, University of Chieti-Pescara, 66100 Chieti, Italy; ascarano@unich.it; 8 Department of Biomedical, Surgical and Dental Sciences, Università degli Studi di Milano, 20122 Milan, Italy; benedetta.morandi@unimi.it (B.M.); massimo.delfabbro@unimi.it (M.D.F.); marco.farronato@unimi.it (M.F.); gianluca.tartaglia@unimi.it (G.M.T.); silvio.taschieri@unimi.it (S.T.); 9 Dental Clinic, IRCCS Istituto Ortopedico Galeazzi, 20161 Milan, Italy; 10 UOC Maxillo-Facial Surgery and Dentistry, Fondazione IRCCS Ca Granda, Ospedale Maggiore Policlinico, 20122 Milan, Italy; 11 SET-118, Department of Pre-Hospital and Emergency-San Giuseppe Moscati Hospital, 74100 Taranto, Italy; mario.balzanelli@gmail.com; 12 Department of Biosciences, Biotechnologies and Biopharmaceutics, Campus Universitario, University of Bari, 70125 Bari, Italy; andrea.ballini@uniba.it; 13 Department of Precision Medicine, University of Campania, 80138 Naples, Italy; 14 Department of Medical-Surgical and Dental Specialties, University of Campania Luigi Vanvitelli, 80100 Naples, Italy; ludovica.nucci@unicampania.it; 15 Department of Oral Surgery, Institute of Dentistry, I. M. Sechenov First Moscow State Medical University, 119146 Moscow, Russia

**Keywords:** SARS-CoV-2 (COVID-19), vaccines, anticoagulants, antioxidants, antivirals, antimalarials, immunotherapy, immunomodulators, corticosteroids, autologous stem cells

## Abstract

Background: Severe acute respiratory syndrome coronavirus 2 (SARS-CoV-2) is the virus responsible for the coronavirus disease of 2019 (COVID-19) that emerged in December 2019 in Wuhan, China, and rapidly spread worldwide, with a daily increase in confirmed cases and infection-related deaths. The World Health Organization declared a pandemic on the 11th of March 2020. COVID-19 presents flu-like symptoms that become severe in high-risk medically compromised subjects. The aim of this study was to perform an updated overview of the treatments and adjuvant protocols for COVID-19. Methods: A systematic literature search of databases was performed (MEDLINE PubMed, Google Scholar, UpToDate, Embase, and Web of Science) using the keywords: “COVID-19”, “2019-nCoV”, “coronavirus” and “SARS-CoV-2” (date range: 1 January 2019 to 31st October 2020), focused on clinical features and treatments. Results: The main treatments retrieved were antivirals, antimalarials, convalescent plasma, immunomodulators, corticosteroids, anticoagulants, and mesenchymal stem cells. Most of the described treatments may provide benefits to COVID-19 subjects, but no one protocol has definitively proven its efficacy. Conclusions: While many efforts are being spent worldwide in research aimed at identifying early diagnostic methods and evidence-based effective treatments, mass vaccination is thought to be the best option against this disease in the near future.

## 1. Introduction

Since December 2019, severe acute respiratory syndrome coronavirus 2 (SARS-CoV-2) has spread rapidly from Hubei province in China to all over the world, causing over 168,996,139 confirmed cases (WHO, 27th May 2021) [1]. More than 3,511,201 million deaths have been caused the coronavirus disease (COVID-19), affecting 192 countries, and these numbers are still growing steadily [2,3]. COVID-19 is an emerging acute respiratory infective pathology that primarily spreads through the airway’s tract by droplets release, as well as respiratory and salivary secretions and direct and indirect contact. Its spread through aerosols (airborne transmission) is suspected to be another important route of transmission, but this is yet to be established [4,5,6]. Some subjects affected by SARS-CoV-2 vector present a viral RNA titer or live infectious virus present in feces, which suggests another possible route through a fecal–oral pathway [7]. According to recent epidemiological data, the incubation period ranges from 1 to 14 days, with an estimated median incubation period of 5.1 days, while transmission could be reported during the pre-symptomatic phase. Moreover, asymptomatic cases represent a considerable percentage of infections, and are likely to contribute to virus circulation [8,9,10,11,12,13,14,15]. Elderly subjects in particular are affected by other comorbidities such as hypertension, cardiovascular diseases, obesity, and diabetes, and subjects with primary or secondary immunodeficiencies have the highest mortality rate [16,17,18,19,20]. Although children tend to experience only mild symptoms, previously healthy young adults have also succumbed to COVID-19 [21,22,23,24]. From the first phases of the COVID-19 pandemic spread, a great amount of information about diagnostic and therapeutic approaches has been reported in the literature, mainly based on preliminary experiences determined by retrospective clinical papers or case reports. Currently, no registered drugs have demonstrated efficacy and safety in the treatment of COVID-19, while several vaccines are available [25,26,27,28,29,30]. Management is mainly based on supportive therapy and on treating the symptoms while trying to prevent respiratory failure. It is fundamental to ensure patient isolation in order to avoid transmission to other patients, family members, and healthcare providers. Quarantine restrictions are necessary to isolate infected subjects at both symptomatic and asymptomatic stages, as well as all persons coming into contact with them [31]. Entire populations must limit social contact and minimize time spent outside [32]. In mild cases, self-isolation at home is the best option, whilst maintaining adequate hydration and nutrition and treating symptoms such as fever, sore throat, or cough. Thus, hospital beds are available for severe cases [33]. Most of the data available for pharmacological treatments come from medications used during the SARS-CoV or MERS-CoV pandemics or from in vitro observations [34,35]. Several clinical trials of possible treatments for COVID-19 are underway, which are based on antiviral, anti-inflammatory and immunomodulatory drugs, cell therapy, antioxidants, and other therapies [36]. There is no evidence that antibiotic prophylaxis can prevent bacterial superinfection, and no evidence of a diagnostic role of procalcitonin in COVID-19 patients. Superinfections are more likely to develop in a hospital environment than at home. Ruan et al. reported a death rate of 16% in COVID-19 patients who had contracted secondary infections [37]. The use of steroids to treat SARS-CoV and MERS-CoV cases was associated with increased mortality, secondary infections, and complications such as psychosis, hyperglycemia, delayed viral clearance, and increased mutation rate of the pathogen. Steroid-based therapy become necessary; thus, it is mandatory to use the lowest possible dosage and only for a short period of time [5,38,39,40,41,42,43,44]. Anticoagulation therapy is recommended in patients with early stage COVID-19, especially when the D-dimer value is four times higher than normal. Infection, inflammation, and other disease-related factors can cause overactivation of coagulation, increasing the risk of augmented ischemic events and disseminated intravascular coagulation [45]. In association with supportive intensive protocols and antiviral administration, the use of immunomodulatory agents and/or convalescent plasma transfusion has been proposed and is currently under investigation by clinical trials. Recent findings in treatment relevance, predictability, and novelty are key factors for an evidence-based service for clinical healthcare operators in order to improve the healing process of COVID-19 subjects by reducing morbidity and any possible adverse effects of the therapies. This manuscript aims at reviewing the current available literature on the main pharmacologic approaches and drugs approved for emergency authorization use, and it also focuses on the innovative clinical protocols for humans and recent advances in the management of COVID-19 patients in clinical human studies.

## 2. Materials and Methods

Using online databases, we carried out a systematic literature review of the treatments for COVID-19 which was restricted to clinical studies. The key articles were retrieved from PubMed (MEDLINE), Google Scholar, UpToDate, Embase, and Web of Science. The keyword search was conducted according the “PubMed Clinical Queries” function and limited to “COVID-19 Articles” category. Our Boolean search is reported in Table 1.

We included scientific publications from the 1st January 2019 to the 1st October 2020. Only publications which focused on treatments for SARS-CoV-2 were eligible for inclusion. We screened all reference lists of relevant studies in order to identify any missing publications. Two independent expert reviewers (RB, FL) performed the article screenings, the title/abstract screenings, and the paper selection. All articles deemed potentially eligible were retrieved for full- text review. We limited our search results to publications in English and excluded abstracts from conferences and commentaries [46]. Some of the drugs approved for other diseases have previously been tried in patients with SARS-CoV and MERS-CoV, and have been evaluated for the treatment of COVID-19 [46,47,48,49]. To date, several randomized clinical trials (RCT) across the globe (https://clinicaltrials.gov/ accessed on 23 May 2021) are investigating such drugs, as well as new compounds. An overview of the main treatments documented so far is given in the following paragraphs.

## 3. Results

**Figure 1:**
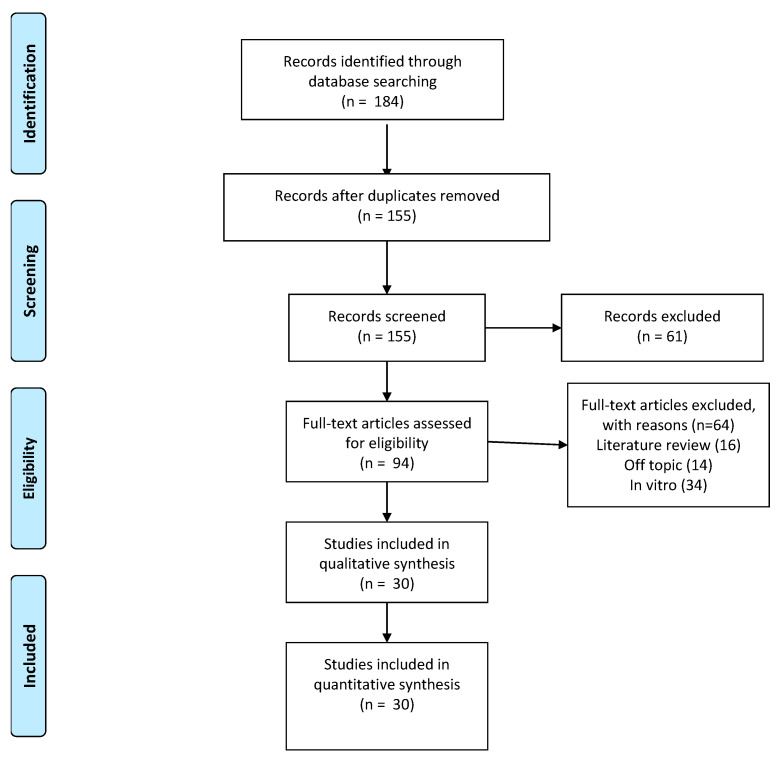
PRISMA flowchart summary of the manuscripts and scientific contribution selection [50].

**Figure 2:**
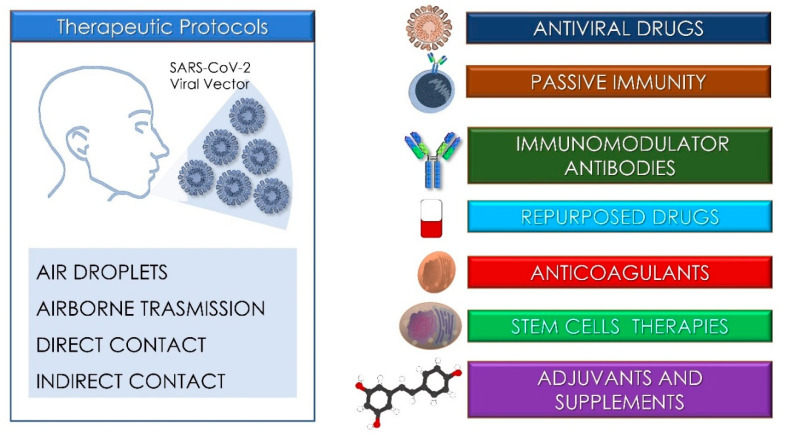
Summary of the therapeutic drug categories proposed against COVID-19.

The research retrieved a total of 155 papers which were identified by an electronic databases search after duplicates were removed. A total of 61 articles were excluded through title and abstract evaluations. The authors’ eligibility assessment excluded a total of 64 papers, while 16 reviews, 34 in vitro studies, and 14 off topic manuscripts were also excluded. A total of 30 papers were included for the qualitative analysis (Figure 1 and Figure 2).

### 3.1. Antiviral Drugs 

The efficacy of specific antiviral agents to treat COVID-19 has been shown both in vitro and in animal models, as well as in anecdotal evidence from human patients (Table 2).

These studies are almost exclusively based on experience with SARS-CoV and MERS-CoV [52,53,61,62,63]. The Italian Society of Infective and Tropical Diseases recommends the administration of antiviral agents to patients with a proven diagnosis of COVID-19 and with mild symptoms [64]. Remdesivir was authorized for the treatment of COVID-19 pneumonia in hospitalized patients, over 40 kg, and >12 years [64]. However, antiviral agents should be avoided in the presence of comorbidities and an increased mortality risk in moderate or severe COVID-19 subjects. Remdesivir was successfully used in several COVID-19 patients in China [65]. It was discovered and synthesized in 2017 by Siegel et al. to counter Ebola and other emerging viruses [66]. Later on, there were different preclinical and randomized clinical studies that analyzed its mechanisms of action and its efficacy against these microorganisms, respectively [61]. It has broad-spectrum antiviral activity against RNA viruses. It is a pro-drug, the structure of which resembles adenosine [66]. It can be incorporated into the nascent viral RNA and inhibit the RNA-dependent RNA polymerase by stopping the replication of the viral genome [67]. Remdesivir has previously been shown to exhibit antiviral activity against several coronaviruses, including SARS-CoV and MERS-CoV, in vitro and in vivo [61,62]. In a recent in vitro study, Remdesivir has also been shown to inhibit SARS-CoV-2 [61,68]. A comparative therapeutic effectiveness of Remdesivir alone and in combination with Lopinavir, Ritonavir, and interferon beta against MERS-CoV has been proposed [62]. Clinical efficacy trials of the use of Remdesivir in COVID-19 patients are currently underway, both in China and the USA. The drug has been administered in COVID-19 patients in the USA and Europe under compassionate use, and anecdotal evidence of benefit has been reported, but no hard data have been produced yet [62]. The current dose under consideration is a single 200 mg, followed by a 100 mg daily infusion for a period of time of 5–10 days. The most common severe adverse effect of Remdesivir is a reversible increase in transaminases, with possible kidney damage [69]. Some randomized clinical trials are ongoing, with the aim to evaluate the effectiveness of Remdesivir administration for the treatment of COVID-19 subjects. The AIFA (Italian Medicines Agency) authorized two randomized, open-label, multicenter phase 3 trials to highlight the efficacy of the Remdesivir antiviral drug compared to supportive protocols (“ClinicalTrials.gov”, n.d.). In this study, it was found that, in hospitalized patients with lower respiratory tract infection, mean recovery time was 10 days vs. 15 days for patients who were given a placebo, while the mortality detected at 15 days was 6.7% against 11.9%, and that at 29 days was 11.4% against 15.2% [70]. Preliminary results from some ongoing studies randomized trials suggest the inclusion of this agent for the treatment of COVID-19. Thus, on the 22nd October 2020, the FDA approved the emergency use of Remdesivir for the treatment of COVID-19 [65,71]. The bovine lactoferrin (BLF), which inhibits viral attachment to the host cell, has a synergistic antiviral effect with Remdesivir against SARS-CoV-2, as manifested in combination therapy studies in cell culture [72,73]. The second-generation antiretroviral drug combination of Lopinavir/Ritonavir inhibits viral protease. Retrospective data from SARS epidemic suggested that an early antiviral treatment with Lopinavir/Ritonavir (LPV/RTV) can potentially reduce the incidence of severe and critical cases [51]. The efficacy of Lopinavir/Ritonavir against SARS-CoV has been demonstrated [52], and these drugs also seem to reduce the viral load in COVID-19 patients [53,63]. However, the clinical evidence for this combination therapy remains limited, as suggested by case reports, while Cao et al. [55] observed no clinical benefit of Lopinavir/Ritonavir compared with the standard of care. The current Lopinavir/Ritonavir dose protocol under investigation consists of doses of Lopinavir (200 mg) and Ritonavir (50 mg) every 12 h for 7–14 days [74]. Although further clinical trials are underway on Lopinavir/Ritonavir, current data do not support Lopinavir/Ritonavir in COVID-19 treatment. According to a recent RCT, about 50% of patients treated with Lopinavir/Ritonavir experienced at least one adverse effect and 14% of patients had to interrupt the therapy [55]. Common side effects of Lopinavir/Ritonavir include nausea, diarrhea, and insomnia, and numerous drug interactions have been reported. Clinical trials that are evaluating other antivirals drugs such as Arbidol, Oseltamivir, Interferon beta-1A, Darunavir and Cobicistat are ongoing, but data are not yet available. Additional studies are needed to evaluate the possible clinical benefits of early use of LPV/RPV in COVID-19. Currently, the manufacturer (Gilead) is transitioning from individual compassionate use requests to an expanded access program for emergency access to the drug for severely ill patients with confirmed COVID-19. Another antiviral used is Favipiravir. It was developed in 2002 and was approved for medical use in Japan in 2014 [56]. In 2018, Favipiravir was also studied as a potential countermeasure against emerging RNA viruses [75]. Therefore, similar to Remdesivir, Favipiravir works as an RNA-dependent RNA polymerase inhibitor which structurally resembles endogenous guanine [57]. Favipiravir is also considered as a novel viral RNA polymerase inhibitor [57]. In mid-March 2020, China announced that Favipiravir had shown good clinical efficacy against COVID-19 [76]. However, it remains to be clarified at what stage of infection with COVID-19 is Favipiravir most effective. For the treatment of COVID-19, doses at the upper limit of the average dosage range should be considered. Appropriate doses of Favipiravir against coronavirus are still under investigation. Some trials are testing protocols with attack doses of 1800–2400 mg, followed by maintenance doses ranging from 300 mg to 1800 mg [58]. Favipiravir is generally well tolerated. However, knowledge about safety in higher dose regimens is limited [48,59,60]. Favipiravir can cause hyper-uricaemia, an increase in transaminases, a decrease in the number of neutrophils, and diarrhea [57]. One study compared Favipiravir with Umifenovir. After seven days of therapy, a significant difference was found between patients treated with Umifenovir or Favipiravir [22], with better results returned by the latter. These data suggest that further clinical trials on the efficacy of Favipiravir for the treatment of COVID-19 are required. Despite little scientific evidence and a limited number of clinical trials available, in March 2020, Favipiravir was approved by the National Medical Products Administration of China as the first anti-COVID-19 drug in China. Another drug of interest is the antiparasitic drug Ivermectin, where in vitro data suggest potential benefits. 

### 3.2. Repurposed Drugs

Ivermectin has been studied since 1946 for use against avid diphtheria, and this molecule was reported as an enigmatic multi-purpose “wonder” drug in 2017 [77]. More recently it has also been used as an anti-parasitic against scabies and equally against HIV, Zika, Dengue, West Nile, and Influenza viruses [78]. Recently, an in vivo study has shown Ivermect ability to reduce viral RNA up to 5000 times after 48 h of SARS-CoV-2 infection [79]. The EMA recommends not to use Ivermectin for the prevention or treatment of COVID-19 outside of clinical trials, while its actions seem to be effective at much higher concentrations than those achieved with currently authorized doses for clinical use [80].

It is currently under study in COVID-19 positive patients, with a dosage of 12 mg per week, together with Hydroxychloroquine (400 mg/day) and Azithromycin (500 mg/day). According to the pharmacokinetic evidence, the dose required to demonstrate in vivo efficacy for COVID-19 treatment in humans would most likely prove to be too toxic to use [81]. All randomized clinical trials published to date conclude that the use of Lopinavir/Ritonavir or Darunavir/Ritonavir or Cobicistat is not recommended for the purpose of preventing or treating the infection because they are ineffective as COVID-19 therapies [51,55,82]. The antiviral activity of antimalarial drugs Chloroquine (CQ) and Hydroxychloroquine (HCQ) has been recently tested [68,70,83,84,85,86,87,88,89] (Table 3).

The results and evidence obtained from the use of Hydroxychloroquine in anti-COVID-19 therapy in hospitalized patients demonstrate an increase in adverse events and a real lack of efficacy [85,88,90,91]. All major scientific societies and international organizations (EMA, FDA, WHO, NIH, IDSA) do not recommend the use of Hydroxychloroquine in hospitalized patients. Any use could be considered in non-hospitalized patients, but only if included in a clinical study trial [92,93]. 

#### 3.2.1. Antibiotics

As a protocol, the routine of antibiotics is never used to treat viral infections or the presence of a viral infection. Antibiotics can be only considered when the symptoms persist beyond 48–72 h and the clinical course suggests a bacterial overlap; for example, if there are radiological signs of pneumonia or in hospitalized patients where there is a risk of superinfection, otherwise the growth of resistant bacteria is increased. The lack of evidence of efficacy in the treatment of patients with viral SARS-CoV-2 infection alone does not suggest the use of antibiotics, either alone or in combination with other drugs, with particular reference to Hydroxychloroquine [94,95]. The RECOVERY trial studied the efficacy of azithromycin (this is a commonly used antibiotic that also has an anti-inflammatory effect), which has also shown some activity against the SARS-CoV-2 virus. The results of the trial showed that there was no benefit in patients treated with this drug; neither the mortality rate nor the length of hospital stay was reduced. There were no reductions in the risk of invasive mechanical ventilation [70]. 

#### 3.2.2. Interferon

Other approaches being pursued include the use of the cytokine interferon (interferon beta-1a), which can be applied by inhalation directly to the lungs, where it can activate the immune response. The Medicines and Healthcare Products Regulatory Agency (MHRA) and Health Research Authority (HRA) [96] have approved a fast-tracked phase-2 trial with interferon in the United Kingdom. It is one of the drugs that can be used either alone or in combinations, such as Lopinavir/Ritonavir, and it has been studied for treating coronavirus diseases [97]. However, IFN-associated adverse effects, cost, and intravenous dosage form would pose major challenges in an outbreak. [98] Nebulized therapy allows the direct release of interferon to the lungs, where it can stimulate the expression of genes that directly participate in viral RNA degradation, interference with viral replication, or assembly. Whether interferon beta-1a has an effect on prolonged symptoms of COVID-19, particularly pulmonary symptoms, will need to be assessed. [98]. Genetically humanizing the immune system of mice produced completely human naturally occurring antibodies [99,100]. Recent studies reported on the ability to separate single B cells from human patients who had previously contracted infection by molecularly cloning the antibody genes produced by B lymphocytes. [101,102,103]. This resulted in independent sources of human antibodies, albeit targeting specific infectious agents. According to these studies, highly effective and performing single antibody pairs were selected and were bound simultaneously, and were not in contrast to the receptor binding domain of the spike protein. [104,105]. By culturing a pseudo virus that manifests the spike in the presence of individual antibodies, scientists can select spike mutants resistant to that antibody. [104]. Monoclonal antibodies are not an alternative to vaccines, but rather a complementary therapy. They did not show important side effects, but they are potentially usable in all subjects [103]. Few of them have reached certain data, including the Regeneron cocktail and Eli Lilly’s drug, currently the only ones on the market. The database of the World Health Organization (https://www.who.int/ictrp/en/, accessed on 16 April 2021) and the National Institutes of Health (https://clinicaltrials.gov/ accessed on 16 April 2021) have identified clinical studies on antiviral mAbs as treatments for COVID-19, starting on 9 November 2020. On 5 February 2021, the AIFA declared the opinion of the Technical Scientific Commission (CTS) and stated that, although it does not have sufficient and mature data on the certainty of the benefits of these therapies, it believes that, in an extraordinary way and in consideration of the situation, it may be appropriate to offer a therapeutic alternative to non-hospitalized subjects with mild/moderate disease who are at high risk of developing a severe form of COVID-19, with a high probability of hospitalization and/or death [106]. On the 8th February 2021, the decree in Italy was published (valid only for 180 days if it is not converted into law), which approves the use of monoclonal antibodies of the companies Eli Lilly/Bamlanivimab and Roche/Regeneron to prevent the onset of severe forms of COVID-19 in patients with mild but at-risk disease [107]. The EMA Committee for Medicinal Products for Human Use (CHMP) is evaluating the available data on the monoclonal antibodies Casirivimab, Imdevimab, Bamlanivimab, and Etesevimab used to treat COVID-19 patients without oxygen therapy support and who are at high risk of severe COVID-19 infection. The committee will initiate two separate reviews, one for Bamlanivimab/Etesevimab blend and the other for Casirivimab/Imdevimab [108]. Initially, the collected data from both studies indicate that the cocktails produced a reduction in viral load (amount of virus in the back of the nose and throat) and led to fewer COVID-19-related doctor visits and hospitalizations. The committee will review the use of Bamlanivimab alone, as early data indicate that monotherapy with Bamlanivimab reduces viral load and promotes a good clinical outcome. The use of Bamlanivimab as a single drug has received authorization from the FDA (U.S. Food and Drugs Administration) and the Government of Canada [109,110,111].

### 3.3. Passive Immunity

#### Convalescent Plasma

Convalescent plasma therapy (CP) has been used for the treatment and prevention of several infectious diseases including the 2003 SARS-CoV-1 epidemic, the 2009–2010 H1N1 influenza virus pandemic, and the 2012 MERS-CoV epidemic [112,113,114], for which modern medicine has no specifically effective treatment (Table 4) [75]. 

Convalescent plasma collected from donors who have survived an infectious disease by producing protective antibodies is considered to provide a great degree of protection for recipients affected by an emerging virus [112]. A significant reduction in viral load and mortality has been shown in studies which use convalescent plasma for the treatment of severe acute viral respiratory infections, such as SARS, MERS, Ebola, H1N1, and H5N1 [113,115,116,118,119], with a significantly better outcome obtained with earlier transfusion [120]. 

**Figure 3:**
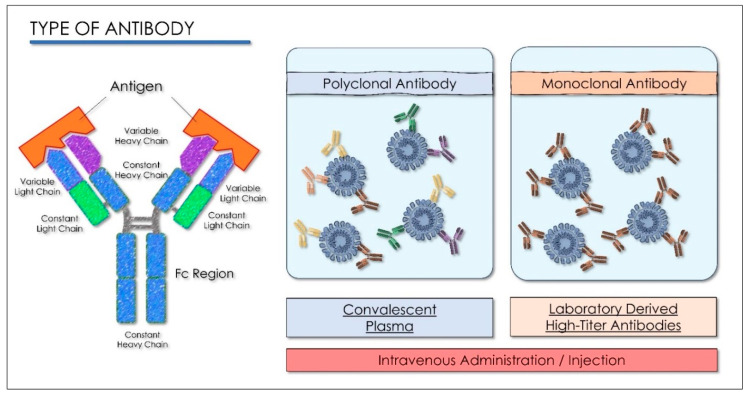
Details of polyclonal/monoclonal characteristics and administration protocols.

**Figure 4:**
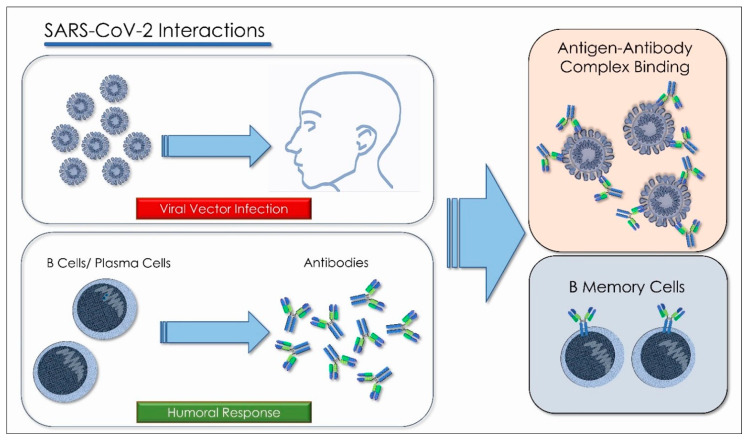
Humoral response of the antigen and human cellular immunity against SARS-CoV-2 infection.

In the absence of a therapy, antiviral drugs, and vaccines specific for COVID-19, many clinical trials have been conducted to investigate the efficacy of convalescent plasma for the treatment of moderate and severe cases of SARS-CoV-2 subjects. A recent Chinese study confirmed the efficacy of convalescent plasma for the treatment of SARS-CoV-2 (Figure 3) [121]. The article reported that COVID-19 patients showed a significant improvement in clinical symptoms after approximately 1 week from the convalescent plasma transfusion. Duan et al. reported that a total of ten subjects affected by severe COVID-19 received an administration of convalescent plasma (200 mL) obtained from recovered patients with neutralizing antibodies titers > 1:640. All patients enrolled in the study met the primary and secondary end points about the transfusion safety, and a significant improvement in clinical symptoms was associated with an amelioration of the radiological and laboratory parameters within 3 days after treatment. All the patients received the standard protocol of antiviral drugs, so these therapies probably also contributed to the desired effect [117]. Given the clinical effectiveness of convalescent plasma, the FDA has granted clinical permission to apply convalescent plasma to the treatment of critically ill COVID-19 patients (FDA, 2020) [122]. An observational study found the usefulness of convalescent plasma for the treatment of COVID-19, showing that 7-day and 30-day mortality was lower in those who received convalescent plasma within three days of the onset of symptoms [117]. Participants in this study were found to have higher antibody positivity and higher neutralizing antibody titers compared to convalescent plasma donors. The reason was assumed to be because most of the donors were young and had only asymptomatic or mild disease [123]. The convalescent plasma exerts neutralizing effects against the virus by reducing the negative time of viral RNA [124]. Unfortunately, the effective titration of antiviral neutralizing antibodies, the targeted timing for treatment with convalescent plasma, the optimal timing for plasma donation, and the severity of the clinical course in patients who might benefit from convalescent plasma remain unclear. (Figure 4) [122]. The donation can be made by subjects with previous SARS-CoV-2 infection and clinical recovery (no symptoms) for at least 28 days, and after at least two NAT tests (nucleic acid test, a test that detects the possible presence of the virus) which are negative for a nasopharyngeal swab and in serum/plasma after 24 h and after healing. The donor must be over 18 and under 65. It is necessary that the serum titer of specific neutralizing antibodies is >160 with the EIA method or with other equivalent methods [125]. The donation excludes women who have had pregnancies, even those not completed, and anyone who has had previous transfusions. This is because antibodies called anti-Hna or anti-Hla develop in these patients, which can cause Transfusion-Related Acute Lung Injury (T.R.A.L.I.), with severe lung damage. This is the main complication causing a serious, sometimes fatal, unwanted reaction to the transfusion [125].

### 3.4. Immunomodulators and Antibodies 

There are many study groups around the world that are working on the development of effective monoclonal antibodies against COVID-19. Neutralizing antibodies have become an important tool in the treatment of infectious diseases. Two separate approaches were worked on which resulted in successful antibody treatments—such as for Ebola; one from genetically humanized mice and the other from an infected and cured (convalescent) human—to generate antibodies against the respiratory syndrome spike protein of acute severe coronavirus 2 (SARS-CoV-2), leading to a large number of fully human antibody species by binding, neutralizing, and three-dimensional structure characteristics.

Passive immunization is the passage of antibodies which occurs physiologically during pregnancy. The transplacental transfer of maternal antibodies to the fetus protects the newborn from many infectious diseases, especially for the first months of life when they are most vulnerable [126]. Most antibody preparations administered to patients contain polyclonal antibodies, derived from immunized animals serum, immunized humans, and sera from convalescent patients (Table 5) [126,127]. 

**Figure 5:**
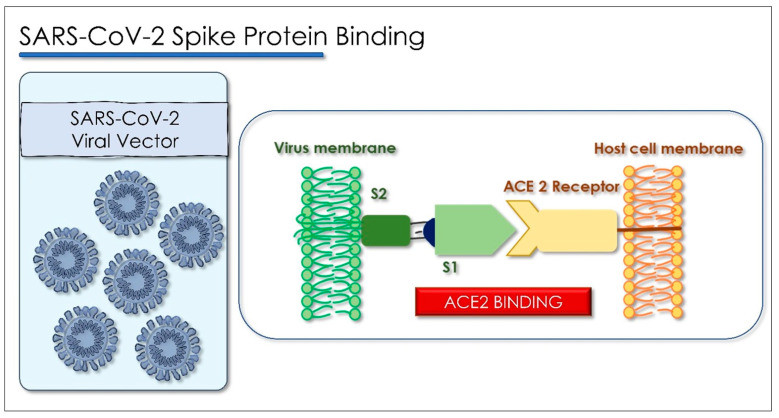
Details of the SARS-CoV-2 S Spike protein (S) binding with ACE2 human receptors.

The production and standardization of the use of polyclonal antibodies associated with patient safety and ease of access have prompted researchers to explore the possibility of replacing polyclonal antibodies with monoclonal antibodies (mAbs). These can be produced through recombinant deoxyribonucleic acid technologies. The use of monoclonal antibodies is applied in the prevention of infectious diseases. MAbs bind in a targeted manner to a specific target in the body. This link is modulable and can be imitated, blocked, or changed to obtain precise mechanisms so that they can arrive at effective therapies with a very specific treatment for targeted diseases. [129,130]. The MAbs produce passive immunization, recognize the epitope region from foreign particles of the virus, and can reduce virus replication and severe disease course [131]. When these antibodies are used in the therapeutic field, the name of the antibody is determined by the therapeutic target and the source from which the antibody is derived, according to the name used by both the World Health Organization, with the International Nonproprietary Names (INN), and from the United States, with the United States Adopted Names (USAN), for pharmaceutical products, and all end with the suffix “MAB”. The suffix “MAB” is related to Monoclonal AntiBody (MAB). As there is a 77.5% similarity between SARS-CoV-2 and SARS-CoV in the amino acid sequence of the spike protein, several studies have suggested the use of SARS antiviral monoclonal antibodies in patients with SARS-CoV-2. Most monoclonal antibodies were targeted to identify the SARS-CoV S1 fragment. The binding domain (RBD) receptor in the S1 subunit is considered the most important target for SARS-CoV-2 because it regulates the adhesion of the virus to the host cell [132], so the mAbs would block the interaction of RBD with its ACE2 receptor (Figure 5) [133]. 

**Figure 6:**
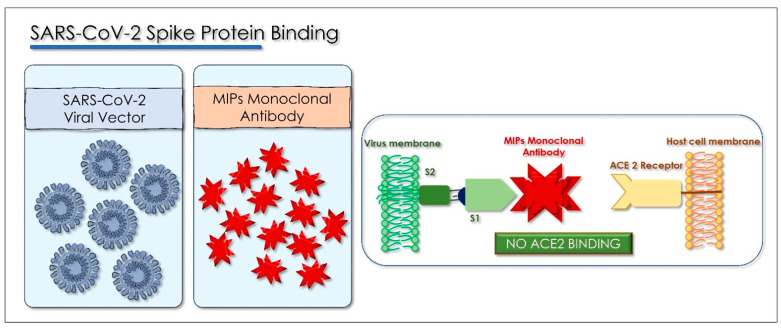
Monoclonal Antibody (MIPs) mechanisms against the SARS-CoV-2 S protein binding with the host cells.

Some monoclonal antibodies recognize epitopes in the S2 unit of SARS-CoV by intervening other mechanisms that neutralize the virus (Figure 6) [134]. The combination of monoclonal antibodies specific for S proteins in SARS-CoV recognizes several epitopes in a laboratory, as well as in vivo cells that may be potentially effective at the viral level; CR3022 has no neutralizing ability alone, but a cocktail of CR3022 and CR3014 showed viral neutralization [135]. Computed tomography (CT) images of patients with acute conditions showed that viral load decreased within days of treatment, while patients’ clinical conditions also improved [136]. More than 40 therapeutic mAbs are currently in use, targeting a range of noncommunicable diseases [126]. Antigens that do not undergo mutations are the most valuable targets for passive immunotherapy, as they reduce both the number of mAbs needed to obtain an effective cocktail and the possibility of escape from the antibody. The great variability of viruses poses a problem for mAb therapy. One way to counter this problem is to use recombinant technology “cocktail” to obtain a specific antibody for an epitope conserved between different viral strains or variants [137]. The results of recent studies have produced new COVID-19 neutralizing antibodies: B38 and H4 of human origin and 47D11 of murine origin. These are new antibodies that have shown excellent results. All act on the S1B receptor binding domain (RBD) of SARS CoV-2 and inhibit binding of protein S to the human ACE2 receptor [129]. A follow-up study showed that the prophylactic administration of mAbs (CR3014) reduced the replication of SARS-CoV in the lungs of infected ferrets, avoided SARS-CoV-induced macroscopic lung disease, and prevented the spread of the virus in the pharynx [135].

#### 3.4.1. Tocilizumab

**Figure 7:**
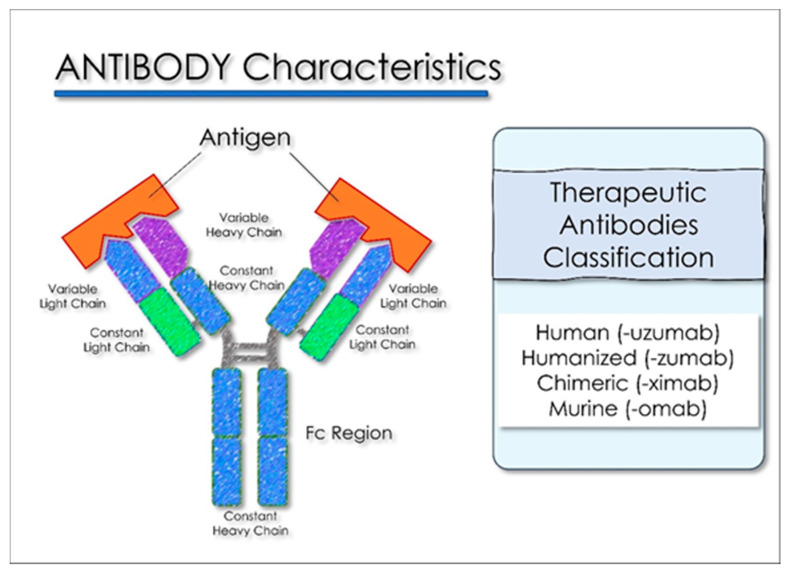
Antibody (MIPs) characteristics and mechanisms of antigen binding.

Tocilizumab is a humanized monoclonal antibody against interleukin-6 receptor (IL-6R Ab), commonly used as an immunosuppressive in the treatment of rheumatoid arthritis and systemic juvenile idiopathic arthritis (Table 6, Figure 7). 

It is currently postulated that patients with severe manifestations of COVID-19 experience some degree of cytokine storm, which results in ARDS and death [37,138,139]. A recent retrospective study evaluated an antiviral administration in 21 subjects affected by COVID-19 for a week who reported persistent fever and worsening of CT tomography evidence and hypoxia; the study reported a potential therapeutic efficacy of tocilizumab for this class of subject. Indeed, after treatment, in addition to the improvement of body temperature, respiratory function, imaging, and lymphopenia improved in most of the patients, with a normalization of inflammatory markers without a significant adverse event [128]. Based on this observation, a multicenter, single-arm, open-label, phase 2 study is currently ongoing but, as with the other drugs discussed here, larger trials are required with appropriate controls before science-based recommendations on use can be made [140]. Recent studies reported the poor efficacy of this drug [141]. The study administered it at an early stage in patients with recently onset COVID-19 pneumonia who required hospitalization, but not invasive or semi-invasive mechanical ventilatory assistance; it showed no benefit in treated patients either in terms of improvement in the clinical course (entry into intensive care) or in terms of mortality [142]. A study was conducted on patients infused with a cocktail of monoclonal antibodies (two fully human neutralizing monoclonal antibodies against the SARS-CoV-2 spike protein cocktail, combined with REGN-COV2) to reduce the risk of emergence of treatment-resistant mutant viruses. In this study, it was found that the REGN-COV2 cocktail reduced viral load, with a greater effect in patients whose immune response had not yet started or who had a high viral load at the start [143].

#### 3.4.2. Baricitinib

Baricitinib is a small-molecule Janus kinase 1 (JAK1) e 2 (JAK2)” inhibitor that is currently approved for treatment of rheumatoid arthritis. AP2-associated protein kinase 1 (AAK1) is a known regulator of endocytosis, and the entry of most of the viruses is dependent on the receptor mediator endocytosis. Hence, the disruption of AAK1 may block the virus’ entry into the cells. Arguably, Baricitinib should disrupt ACE2-mediated SARS-CoV-2 endocytosis entry into cells within the same therapeutic plasma concentration range when used for rheumatoid arthritis; therefore, Baricitinib is postulated to be another candidate for clinical trials to treat COVID-19 [120,144,145]. The study showed that patients administered with the Baricitinib–Remdesivir combination therapy improved the clinical course faster and were 30% more likely to improve on day 15 than patients on monotherapy. There was a lower rate of serious adverse events (16% vs. 21%). The efficacy of Baricitinib–Remdesivir treatment was better in severe patients with high oxygen flow or with non-invasive ventilation). In these patients, mean recovery time was 10 days compared to 18 days in the monotherapy group, with greater likelihood of improvement in clinical status [145].

#### 3.4.3. Regen-Cov

The regen-CoV is a mix of human monoclonal antibodies produced by the American multinational Regeneron/Roche which was obtained for emergency use by the American body for the control of drugs from the Food and Drug Administration (FDA) in November 2020. The mixture of REGN10987 and REGN10933 antibodies, recently named Imdevimab and Casirivimab, respectively, is administered together for the treatment of mild to moderate COVID-19 in adults and pediatric patients (12 years of age and older weighing at least 40 kg) who have tested positive for SARS-CoV-2 and who are at high risk of developing severe COVID-19. Additionally, included are those who are 65 or older or those who have a chronic illnesses [109]. The data show that this simultaneous administration is most effective in seronegative subjects with high viral load and with at least one risk factor, and that, in the general population, the rate of infection on day 29 is reduced by approximately 3%. From the data, the percentage of protection is present and increased in subjects at risk [106,146]. As per the opinion of the AIFA CTS on monoclonal antibodies, patients hospitalized for COVID-19 who receive oxygen therapy are excluded. High-risk subjects are characterized by the following factors: BMI ≥ 30, chronic kidney disease, uncontrolled diabetes, primary or secondary immunodeficiencies, age > 65 years; age ≥ 55 years with: cardio-cerebrovascular disease (also hypertension with lesions organ), COPD, and/or other chronic respiratory diseases; patients aged 12–17 years with: BMI ≥ 85th percentile for age and gender, sickle cell anemia, congenital or acquired heart disease, neurodevelopmental disease, constant presence of technological device for assistance in maintaining vital signs (e.g., subjects with tracheostomy, gastrostomy, etc.), asthma, or other respiratory diseases that require daily medical assistance. The methods of administration are by intravenous infusion over a period of 60 min (followed by another 60 min of observation for any serious adverse reactions) [146]. A mixture of fully human antibodies is obtained from both genetically modified mice and B cells of convalescent patients [147]. The REGN10987 and REGN10933 bind, while simultaneously non-competing, to different RBD epitopes of the SARS-CoV-2 spike, preventing binding of the viral protein with ACE2 and thus leading to virus neutralization. The antigen-binding fragment of REGN10933 binds at the top of RBD, removing the binding site for ACE2 from the virus, while REGN10987 acts on the side of RBD, which is less likely to interfere with ACE2. The results indicate that Regen-Cov reduces viral load by reducing the risk of contracting the infection by 50% [104,148,149].

#### 3.4.4. Bamlanivimab

Bamlanivimab is the investigational monoclonal antibody (LY-CoV555) authorized for emergency use (EUA) by the FDA for the treatment of high-risk patients with mild to moderate COVID-19 in the United States and other countries. This drug, manufactured by Eli Lilly and Company, showed a 72% efficacy for reducing the risk of hospitalization for patients with mild or moderate symptoms in studies. In addition, a study by the National Institutes of Health (NIH) in the US showed that the drug could prevent infection in about 80% of residents and staff in nursing homes. The drug should be infused over 1 h in a facility equipped for the management of anaphylaxis. Bamlanivimab was not helpful in hospitalized patients [110,150,151]. The AIFA Technical Scientific Commission clarifies that, to date, emergency use has been authorized (in the USA and Canada) only for the drug Bamlanivimab at a dosage of 700 mg, and that the data relating to the higher dosages of this drug or mixture with Etesevimab are not currently available [110]. The analysis is based on the data available for the 700 mg monotherapy dose of the drug Bamlanivimab. In particular, in non-hospitalized patients with mild/moderate symptoms, this dosage produces a reduction in hospitalizations and/or visits to the emergency room on day 29 after administration in about 5% in the general population and up to 11% in high-risk patients. The company will still have to assess whether there is a correlation of these outcomes with the reduction in viral load, which is not currently documented [106]. 

#### 3.4.5. Bamlanivimab/Etesevimab

This is a mix of monoclonal antibodies produced by Eli Lilly, whose studies have shown that it can reduce the risk of hospitalization and death from COVID-19 by 70%. Bamlanivimab and Etesevimab were tested in COVID-19 positive high-risk patients. The results show that they are also able to reduce viral load and accelerate healing, with a resolution of symptoms [150].

#### 3.4.6. AZD7442

This is a mix of two human monoclonal antibodies (COV2-2196 and COV2-2130) with a Long Acting AntiBody (LAAB) that mimic natural antibodies and has the characteristic of treating and preventing the worsening of the clinical course of the disease in patients potentially infected with the virus. Both COV2-2196 and COV2130 showed strong neutralizing activity against the SARS-CoV-2 strain, and also against recent variants, including E484K, N501Y, and D614G. Produced by Astrazeneca, the same pharmaceutical company that developed the COVID-19 vaccine together with the University of Oxford, it can be used as a preventive intervention in dangerous environments such as communities, hospitals, nursing homes, and student residences, as one of its advantages is that it provides immediate antibodies. It is still under study and it is hoped that it will be available between March and April 2021 [152].

#### 3.4.7. MabCo19

The monoclonal antibody discovered in a laboratory in Siena was the result of research conducted by Prof. Rino Rappuoli. This MabCo19 research project was developed in collaboration with the Lazzaro Spallanzani Institute of Infectious Diseases and was based on the discovery and development of three human monoclonal antibodies against SARS-CoV-2 coronavirus, isolated from the blood of convalescent or recovered patients for prophylactic/therapeutic purposes. It is used as a molecular bait to detect antigens for the production of a future vaccine [153,154].

#### 3.4.8. VIR-7831

On March 11, Vir Biotechnology and GlaxoSmithKline announced that VIR-7831 (GSK4182136), a monoclonal antibody for the initial treatment of patients at high risk of hospitalization [129,147], following safe efficacy data found by an independent data monitoring committee (IDMC), interrupted the enrolment of phase 3 of the COMET-ICE study (COVID-19 Monoclonal antibody Efficacy Trial—Intent to Care Early). Vir and GSK now plan to apply immediately for Emergency Use Authorization (EUA) in the United States and authorization in other countries. VIR-7831 alone demonstrated an 85% reduction in hospitalization or death in patients. VIR-7831 was well tolerated. During phase 3, part of the COMET-ICE study evaluated the safety and efficacy of a single intravenous infusion of VIR-7831 (500 mg) [155]. 

#### 3.4.9. ANAKINRA

The monoclonal antibody Anakinra, from the Swedish company Sobi, works by blocking the cytokine storm responsible for the serious and deadly outcomes of COVID-19 [147,156,157]. The research investigated, for the first time, the efficacy of two different types of anti-inflammatories on patients with severe forms of COVID-19: the interleukin IL-1 inhibitor, called Anakinra, and the IL-6 inhibitors Tocilizumab and Sarilumab. The results of the study found that Anakinra resulted in a 72% reduction in mortality at higher dosages vs. 50% at standard dosages. This showed that the cytokine to be targeted is precisely IL-1, in addition to the need to intervene in a timely manner, since the patients treated before (when the indicators of the inflammatory state were lowest) were also those who had the best prognosis. The cytokines most involved in the inflammatory process are IL-1 and IL-6. The first study attempts focused on the inhibition of IL-6, especially through the administration of Tocilizumab. However, the results were not encouraging [158]. According to the results of the study, only Anakinra produced a substantial reduction in mortality, since the cytokine to be targeted is precisely IL-1 [158].

#### 3.4.10. Corticosteroids 

Corticosteroids were quite commonly used during the previous two SARS and MERS outbreaks (Table 6) [159,160]. 

In both previous outbreaks, histology revealed inflammatory changes and diffuse alveolar damage associated with the infection [49]. Hence, corticosteroids might play a role in suppressing the inflammation but, in addition, they can also hinder the immune response and clearance of pathogens [39]. Although the use of corticosteroids in SARS-COV-2 viral pneumonia is not clearly recommended [39], in case of signs of an exaggerated immune response or in patients with symptoms of myocardial involvement, the use of moderate dosage of corticosteroids for a short time (0.5–1 mg/kg/day/intravenous/intramuscular of prednisone equivalent for 5–7 days) is suggested [162,163]. There are few randomized clinical trials with reliable data that demonstrate the certainty of the efficacy of the use of corticosteroids in the initial stages of the COVID-19 pandemic. Many guidelines on COVID-19 treatment, including those of the WHO, the National Institutes of Health (NIH, USA), the European Society of Intensive Care Medicine, and Society of Critical Care Medicine (ESICM/SCCM), indicated the use of cortisone; not routinely, but only for those patients in refractory shock or those who were already taking corticosteroids for pre-existing chronic diseases before the diagnosis of COVID-19 [164]. The ESICM (European Society of Intensive Care Medicine) guidelines indicate the use of corticosteroids for patients with COVID-19 ventilated with the aid of machines and ARDS (Acute Respiratory Distress Syndrome). The EMA indicates the use of dexamethasone in ages > 12 and weighing > 40 kg and in need of supplemental oxygen therapy (both classical oxygen therapy and mechanical ventilation). The methods of administration of dexamethasone are orally, i.m., or infusion (drip) into a vein. In all cases, the recommended dose in adults and adolescents is 6 mg once daily for up to 10 days. [92]. WHO does not recommend use in subjects with non-serious pathology, but recommends the use of cortisone in subjects with severe or critical cases of COVID-19. The Infectious Diseases Society of America (IDSA) and Centers for Disease Control and Prevention (NIH-CDC) recommend the administration of systemic corticosteroids in subjects with severe COVID-19 disease: dexamethasone (at a dose of 6 mg IV or for 10 days, or until discharge, if earlier) or an equivalent glucocorticoid (e.g., methylprednisolone 32 mg and prednisone 40 mg or hydrocortisone 160 mg, as suggested by the IDSA and AR-CHMPA guidelines). It does not recommend use in patients hospitalized with COVID-19 with a good level of saturation without oxygen therapy support. The circular of the Italian Ministry of Health on the 30^th^ of November 2020 indicates the use of cortisone at home in those patients whose clinical picture does not improve within 72 h in the presence of parameters that require oxygen therapy, as well as in the presence of worsening pulse oximetry parameters which require oxygen therapy [165]. An individual benefit/risk assessment should be considered prior to administering corticosteroid therapy by monitoring likely adverse events. Situations at risk may be: nonspecific ulcerative colitis with a danger of perforation, abscesses or other pyogenic infections, diverticulitis, recent intestinal anastomosis, active or latent peptic ulcer, renal insufficiency, hypertension, osteoporosis, myasthenia gravis, or glaucoma [165]. The use of cortisone in the initial phase of the disease (in which phenomena related to viral replication prevail) could have a negative impact on the immune response in progress [165]. There are insufficient data to evaluate the interactions between Remdesivir and co-administered corticosteroids [92]. 

### 3.5. Anticoagulants 

Disseminated intravascular coagulation (DIC) is also frequently reported in the mild and early stages of the disease [138], and it is strongly associated with a significantly higher mortality (71.4% of non-survivors—0.6% of survivors) (Table 7) [166]. 

SARS-CoV-2 is probably more prone to inducing DIC, also thanks to the hyperimmune host response [45,168]. Tang et al. (2020) investigated the effects of anticoagulant heparin in patients with severe COVID-19 [167]. D-dimer, prothrombin time, and age were positively correlated, and platelet count was negatively correlated, with 28-day mortality. There was no difference in 28-day mortality between heparin users and nonusers. However, the 28-day mortality of heparin users was lower than nonusers. This study suggests that anticoagulant therapy with heparin appears to be associated with better prognosis in severe COVID-19 patients [167]. According to Galluccio et al. [169], heparin is recommended at initial doses of 50 UI/Kg or 25 UI/Kg in patients with bleeding or platelet count < 50 xl 09/L, with aPPT of 40–60 s as a target for anticoagulation maintenance dosage.

In nature, many pathogens use peri- and extracellular glycosaminoglycan heparan sulphate (HSPG) as an adhesion element and then invade the cell subsequently [170,171]. Data from recent studies have revealed that HSPGs, in addition to ACE2, also play a decisive role in the mechanism of adhesion to cells by SARS-CoV-2. Binding of the viral spike to the HSPGs results in an increase in viral concentration in situ, ready for subsequent specific binding with ACE2. It follows that, if interactions between the viral spike protein and the HSPGs are blocked, virus replication is inhibited. Heparin, an anticoagulant drug widely used as an anticoagulant, is similar in structure to HSPG. [171] Based on these structural similarities, studies have been oriented and show that heparin is able to prevent infection by a number of viruses, including SARS-CoV-2; for the latter, it prevents up to 80% with doses of prophylaxis. The binding domain of the spike protein receptor at subunit 1 (S1) (S1 RBD) of SARS-CoV-2 by binding to heparin and enoxaparin (low molecular weight heparin)) induces a secondary structural change in the RDB. The mechanism of inhibition of viral infection is determined by an overlap between the binding sites of heparin/HS on S1 RBD and those of ACE2 (angiotensin 2 converting enzyme) [171,172,173]. The mechanism of inhibition of binding of RBD to human ACE2 protein by binding of heparin to spike S1 RBD protein was investigated using ELISA assays and differential scanning fluorimetry. [173,174]. Until recently, heparin (administered systemically) was only used in COVID-19 patients as an anticoagulant to prevent thrombus formation. In the UK, Brazil, and Australia, studies are being developed on nebulized unfractionated heparin (UFH) that is being tested in COVID-19 patients as a possible treatment. The study involved seven different heparin preparations, including UFH and low molecular weight heparin (LMWH), of porcine or bovine origin, and evaluated their antiviral activities against SARS-CoV-2. All the results found that LMWH is ~150 times less inhibitory than UFH and that heparin binds to the RDB protein. It also directly inhibits the binding of RBD to the human protein ACE2 receptor [174]. The lactoferrin (LF) also prevents viral adhesion to host cells through its antiviral action by binding to HSPGs. In vitro, premixing LF with heparin reduced the inhibitory activity of LF on a viral attack by reducing the antiviral activity of LF. LF, in fact, binds directly to heparin because it is an HSPG camouflage [72,73,170].

### 3.6. Stem Cells Autologous and Allograft

COVID-19 respiratory infection will also probably cause long-term damages to lungs, kidneys, the heart, the nervous system, and the liver. In this respect, the use of mesenchymal stem cells (MSCs) may reveal more than a great tool in contrasting the aggressiveness of the virus, as it could also play a key role in attenuating both the present and long-term side effects of the disease (Table 8). 

This ability is due to the unique biological features of MSCs being capable of regulating the immune system via the stimulation of immune-modulatory cells such as gamma/delta T lymphocytes (γ/δ T Cells), macrophages type 2 (M2), and Th2 T-cells. In addition, MSCs differentiate into mature cells for the formation of new tissues such as the type II alveolar epithelial (ATII) cells that form approximately 60% of the pulmonary alveolar epithelium [175,180,181,182]. By inhibiting the production of pro-inflammatory cytokines and interleukins, such as TNF-a, IFN-y, IL-6, IL-2, and IL-1 via the inhibitory mechanism exerted on monocytes differentiation towards T-lymphocytes, B-lymphocytes, NK cells, and dendritic cells (DCs), the MSCs are shown to mitigate self-destructive auto-immune mechanisms [139,183,184,185,186,187,188]. The MSCs’ ability to limit self-attacking responses is consistent with their low level of co-stimulatory factors CD-80 and CD-86 (MHC I), which are essential for the MHC class II activation and for the triggering of T cells’ second immune response; this is similar to what happens during a severe graft-versus-host disease (GVHD) and during the last phase of COVID-19 infection. In vivo, MSCs have shown they can treat steroid-resistant GVHD in patients who underwent either allogeneic organ or hematopoietic stem cells transplantation, thus improving the general outcomes of the consequences of aberrant auto-destructive immune responses [175,184,189,190,191,192]. Of note, the MSCs’ long telomeres allow them a continuing replicative process, and therefore a constant immune modulation during inflammation and infections. The MSCs’ high number and their fast turn-over may contribute to elucidating their therapeutic rationale vs. Sars-CoV-2. Sars-CoV-2 fast tropism would become the virus’ strongest weakest point. This means that, the faster the tropism, the weaker the virus defenses become, and the faster the tropism, the higher its vulnerability to the MSCs’ elevated concentration and fast renewing rate [175,193]. Stem cells therapies have been proposed for severe acute respiratory syndrome coronavirus 2 (SARS-CoV-2) in moderate and severe hospitalized subjects [176,177,178,179]. In humans, umbilical cord-mesenchymal stem cell (UC-MSCs) infusion has produced a significant decrease in inflammatory cytokines and respiratory parameters, with an early response [177,178,179].

### 3.7. Adjuvants and Antioxidants 

An unclear aspect of treatment is the post-viral syndrome of COVID-19, which afflicts another category of patient defined as “long haulers”, who are not hospitalized subjects, but their healing period is delayed [194] (Table 9). 

This type of patient with a mild or moderate infection, and never hospitalized after 8 or 9 months from the initial infection, fails to heal, and is incapable of recovery after being negative in tests [198,199]. The presence of shortness of breath (SOB) is common in these patients, which causes fatigue after any type of physical activity and effort and reduces work capacity. The constant chronic symptoms are chest and heart pains, intestinal disorders, headaches, difficulty concentrating, memory loss, and tachycardia [200,201]. The chronic symptoms developed, lasting weeks or months, are very similar to myalgic encephalomyelitis/chronic fatigue syndrome (ME/CFS), and are associated with other viral infections, such as Epstein Barr or glandular fever. Many studies lead to Mythondrial dysfunction and oxidative stress in ME/CFS. Therefore, therapeutic approaches that tend to improve mitochondrial health could alleviate both this complex disease and the ailments of patients with chronic post-COVID-19 syndrome. ME/CFS patients who took methylphenidate (Ritalin^®^) with vitamins and minerals improved mitochondrial activity and reported less fatigue [195]. The administration of sodium Dichloroacetate, a pyruvate dehydrogenase/glycolysis inhibitor, also showed an improvement in the feelings of tiredness and fatigue [196]. The relatively high percentage of SARS-CoV-2 infected “long haulers” who do not recover directly in the post-viral period of their disease is due to the damage caused by the host’s response to the initial infection. An important body response, such as a cytokine storm, can cause oxidative and inflammatory damage, such as oxidative stress in general. Antioxidant therapies, such as quercetin, astaxanthin, luteolin, glycyrrhizin, lactoferrin, hesperidin, bromelain, and curcumin, are able to raise the levels of the natural antioxidant, glutathione (which is important for redox balance), which improves the immune response and COVID-19 symptoms [5,19,202,203,204,205,206,207,208,209]. Other clinical studies evaluated N-acetylcysteine, an effective therapeutic agent, to improve the redox state, to replenish glutathione reserves, and to increase the proliferative response of immune T cells [210]. A new clinical trial has already begun in Wuhan, China, evaluating the infusion of vitamin C for the treatment of severe pneumonia in those infected with SARS-CoV-2 [197]. According to the research group of Prof. Alessandro Santin, head of the research team of the Smilow Cancer Center and director of the oncology department of Yale School of Medicine, through the latest data collected, these patients have not suffered major damage to various organs from the cytokine cascade that characterizes COVID-19 [194]. In some organs of “*long haulers*”, who are usually not infectious, the virus would remain in small quantities. As such, the immune system detects its presence, inducing mast cells and macrophages to cause a more controlled inflammatory reaction, producing “*a rain*” and not “*a storm*” of cytokines, which is a sign of persistent chronic inflammation that causes difficulty in healing and asthenia (Rockefeller University study) [211]. These patients are misdiagnosed as depressed, anxious, and hypochondriac (especially in female patients), and then treated with anxiolytics [198]. Due to the persistence of the vital virus, in the case of immune deficiency, these patients could reinfect themselves. An interesting work by Larry Afrin, scholar of mast cells, has shown that, by using antihistamines in these “*long haulers*”, their symptoms improved, along with their quality of life [212]. It is estimated that over 30% of patients infected in a mild and/or moderate form may belong to this type of patient [194].

#### 3.7.1. Lattoferrin 

Lactoferrin (LF) is a glycoprotein present in human, bovine, murine, and porcine species which consists of about 690 amino acid residues belonging to the transferrin family. It is capable of reversibly binding to two Fe atoms per molecule with high affinity, maintaining the bond up to pH values of 3.0 (transferrin retains iron at a pH of about 5.5) [213,214]. This property is important because it ensures the sequestration of iron in infected tissues where the pH is commonly acidic [215]. At the same time, its ability to bind iron limits the availability of essential iron to microbes [215]. The high binding affinity with iron means that, in the presence of LF or transferrin, the concentration of free iron in body fluids is very low (10–12 M). This then prevents the precipitation of iron as insoluble hydroxides, which results in the inhibition of microbial growth and the hinderance of the formation of reactive oxygen species. [73]. It intervenes in regulating the immune response and in the defense mechanisms against bacteria, fungi, and viruses. By acting on cell receptors, LF prevents viral anchoring, surface accumulation on the host cell, and virus entry into the cell itself [216,217]. Its antiviral activity, both on naked and enveloped viruses, occurs in the early stages of infection, preventing the virus from entering the host cell [73]. Since it intervenes in the destruction of cell membranes, in the sequestration of iron, in the inhibition of the adhesion of the pathogen to host cells, and in the formation of biofilms, it prevents the growth of many pathogens [73]. In breast milk, its peak concentration is found in colostrum (8 mg/ml), while lower levels are found in breast milk (3.5–4 mg/ml) and even lower levels in exocrine secretions and secondary granules of mature neutrophils. In the presence of infection and/or inflammation, due to the recall of neutrophils, the concentration of lactoferrin increases [73,218,219]. In premature infants, prolonged administration of milk with higher concentrations of lactoferrin would reduce the high risk of sepsis from the intestinal and respiratory tracts [220,221]. 

In the mechanism of viral infection, particularly in COVID-19, the first phase consists of identifying the first cell anchoring sites. LF can prevent viral infections by interacting with these cell receptors, recognized in those of glycosaminoglycan heparin sulfate (HSPG) [170]. Lang et al. found that LF inhibits infection by occupying the virus anchor sites provided by HSPGs, not allowing preliminary adhesion between SARS-CoV-2 and host cells. The blockade of lactoferrin on the binding mechanism between the viral spike protein and the HSPGs is independent of what occurs on the angiotensin converting enzyme 2 (ACE2) receptor [222]. In fact, after the first anchoring of the viruses on the cell surface, other specific receptors for the viruses are identified and allow the real entry into the host cell. This mechanism of LF, interfering in the viral anchoring, prevents the subsequent phases that allow the viral concentration on the cell surface, the recognition, and the subsequent binding with the specific input receptors; that is, the ACE2, which then determines the real infection [170,223].

Lang et al. studied and tested these effects and mechanisms on SARS CoV and not SARS CoV-2. However, since SARS-CoV and SARS-CoV-2 have 72% identical sequences, and their binding domain structure of the receptor is very similar, these data could be considered [216,222].

All studies have shown that SARS-CoV-2 is mainly transmitted through respiratory droplets, but it can also attack enterocytes that cause gastroenteritis symptoms and can act as reservoirs at the same time. Gastrointestinal symptoms, in fact, are the main clinical sign in newborns [224,225,226,227,228,229,230,231,232,233,234]. 

Colostrum, milk from the first months and lactoferrin, can therefore create a favorable intestinal microbiota with anti-inflammatory action, and which stimulates and strengthens the innate immune defenses of newborns [73]. 

Since iron homeostasis disorders are related to high levels of IL-6, lactoferrin is being considered as a potent regulator of iron homeostasis and inflammation [218,219].

The antiviral activity of lactoferrin, through its binding with host cells or viral particles, or both, confirms the concept that this glycoprotein is an important reinforcing brick of the mucous wall which is effective against viral attacks [73]. 

The massive release of cytokines related to COVID-19 infection significantly affects platelets because platelets have many receptors where these inflammatory molecules can bind and hyperactivate them [188,235,236,237]. This mechanism will produce thrombocytopenia and coagulation phenomena [235].

The administration of lactoferryn is able to reduce the inflammation response, to prevent a thrombocytopenia state and a hypercoagulation reaction by contrast to the entry of the viral vector [217].

Lactoferrin in enteric formulation is able to resist the enzymatic activity of stomach pepsin agents and, at 10 times higher concentration in the circulation compared to lactoferrin, could be considered an important neutraceutical which is potentially effective for prophylaxis and therapeutic administration in COVID-19 subjects [238,239,240].

#### 3.7.2. Vitamin D

Calcitriol (1,25-dihydroxyvitamin d3), as an active form of vitamin D, has antioxidant, anti-inflammatory, and immunomodulating properties, with an increased risk of respiratory tract infections associated with its deficiency [241]. It has been found that the population group most vulnerable to COVID-19 is the older population, and also the one with the most vitamin D deficient levels (the lowest vitamin D levels in the aging of the population are found in Spain, Italy, and Switzerland) [242,243]. The level of vitamin D correlates negatively with the severity of the radiological findings, COVID-19 severity, and mortality, and it could also play a key role as an adjuvant to improve the recovery from moderate and severe diseases [244]. A recent meta-analysis of several studies by Nurshad Ali [245] demonstrated the role of vitamin D in reducing the risk of acute viral infections of the respiratory tract and pneumonia through direct inhibition of viral replication or through anti-inflammatory or immunomodulation action [246,247,248]. The antiviral mechanisms of vitamin D are due to the reduction in the synthesis of pro-inflammatory cytokines and the prevention of the infiltration of immune cells into the lungs [249,250].

Vitamin D exerts effects on the ACE2/ANG (1–7)/MasR axis and improves ACE2 expression so it can be used to decrease lipopolysaccharide-induced acute lung injury through the renin–angiotensin mechanism [243,247]. The degree of RAS overactivation is associated with a worse prognosis. Low vitamin D levels lead to higher RAS activity and higher angiotensin II concentrations [244,249]. 

Many authors reported that vitamin D deficiency is able to reduce the maturation capability of macrophages, decrease macrophages’ production of surface antigens, and inhibit lysosomal phosphatase synthesis and hydrogen peroxide that is correlated to a antimicrobial activity [242,247]. The overlap of the risk factors for severe cases of COVID-19 infection and concomitant comorbidities, such as vitamin D deficiency, obesity, chronic kidney disease, and being of a black or Asian origin, suggests that vitamin D supplementation could provide an important role in prophylaxis and the therapy of the SARS-CoV-2 disease [205,251]. The comorbidities, along with vitamin D deficiency, increase the risk of serious COVID-19 events such as hypertension, diabetes, and metabolic syndrome [244]. Therefore, vitamin D deficiency certainly has an indirect effect on the mortality of COVID-19 (favoring comorbidities) [244]. Moreover, in vitro papers reported that calcitriol, which represents the active molecular form of vitamin D, is associated with antiviral activity against the viral vector of SARS-CoV-2. An important effectiveness from a rat study reported that the vitamin D is able to decrease lipopolysaccharide-induced acute lung damage according to the renin–angiotensin system (RAS), which plays a determinant role for the pathogenetic mechanism related to the SARS-CoV-2 disease, while the degree of RAS hyperactivation is related to a worse prognosis. Low vitamin D levels are correlated with an increased RAS response and augmented angiotensin II concentrations. According to this effectiveness, Vitamin D 3 doses of 2000 IU/day or 4000 IU/day are recommended [19,252,253,254].

#### 3.7.3. Melatonin

Melatonin (N-acetyl-5-methoxytryptamine), secreted mainly by a small gland in the brain called the pineal gland or epiphysis, is a fat-soluble hormone in water. The production of melatonin has a low concentration in the first months of life, increasing at a young age, and then decreasing in old age [255]. Melatonin has been studied due to its anti-inflammatory characteristics and antioxidant properties that could be exerted by different molecular signaling and pathways [256,257]. Melatonin induces the increase and maturation of NK cells, T and B lymphocytes, monocytes, and granulocytes in both bone marrow and other tissues [182,258,259,260,261,262,263,264,265,266]. In the literature, several articles reported that the administration of melatonin is able to provide an increase in the quantity of macrophages/monocytes and an over-expression of antigenic receptors of the single cell/macrophage lineage [267]. Moreover, in the case of an inflammatory process, melatonin is able to produce a decrease in the proinflammatory cytokines release, such as TNF-α, IL-1β, IL-6, and IL-8 and, consequently, an increase in the anti-inflammatory cytokines such as IL -10. Therefore, melatonin administration decreases the levels of the highly sensitive C reactive protein and inhibits the nuclear factor NF-κB [255,256]. Associated to melatonin administration, several authors reported a reduction in the risk of ARDS (acute respiratory distress syndrome), and therefore of a less severe chance of clinical manifestations and mortality. Melatonin was studied due to its ability to decrease the risk of hemorrhagic shock associated with viral infections [268,269]. Moreover, melatonin is able to inhibit the calmodulin release associated with an increase in the expression of ACE2 receptors, which represents the host cell’s elective receptor for SARS-CoV-2 and increases the binding to the cell surface [270]. Melatonin compounds also intervene by the inhibition of an another cell receptor for SARS-CoV-2, i.e., the CD147, which is associated with the regulation of cellular chemotaxis and inflammation of the lung [271]. Associated with lung disease, the ACE2 and CD147 receptors are involved in the regulation of vascular permeability related to the genesis of a pulmonary edema. These receptors are able to activate the renin–angiotensin–aldosterone system (RAAS) and produce severe damage to the lung tissues [272]. The effectiveness of several in vitro articles reported that melatonin inhibits the major protease SARS-CoV-2 (Mpro), an enzyme always present among coronavirus species. Moreover, melatonin is a MPro inhibitor and could represent a broad spectrum SARS-CoV-2 drug [273]. In addition, the bats that represent the main reservoirs of coronavirus produce high levels of melatonin and almost never exhibit the symptoms associated with viral infection [274]. In humans, infants produce higher levels of the melatonin hormone compared to adult subjects, and this aspect could produce a possible contribution to mild clinical manifestations and symptoms in the early age group [275,276]. By the administration of exogenous melatonin compound, there was an increase in the production of antibodies, both in physiological and pathophysiological conditions [277]. Melatonin is considered as an immunomodulator agent. Moreover, melatonin produces an effective stimulation of the immune system in cases of the suppression of the immune response, and it is immunosuppressive when a non-invasive inflammation state occurs [256,278]. In fact, associated with the anti-inflammatory and pro-inflammatory characteristics of melatonin, this molecule is associated with antidepressant, anxiolytic, neuroprotective, and antihypertensive capabilities that are able to ameliorate the clinical healing of an affected patient. In consideration of the previously described factors, this agent can be administered as a prophylaxis and/or treatment in COVID-19-infected subjects [279].

#### 3.7.4. Lianhuaqingwen (Lhqw)

Traditional Chinese Medicine (TCM) has proposed different treatments against COVID-19 infection, both for the prevention and control of the spread of its viral diffusion [280]. A TCM molecule was the Lianhuaqingwen (LHQW). Proposed in a capsule format, LHQW contains a combination of natural extracts from plant-based medicines composed of polyphenols, triterpenes, anthraquinones, iridodiades, more than 12 types of plants, and 61 active agents with previously reported positive effects. LHQW compounds are packaged in accordance with the Chinese Pharmacopoeia [281]. The National Health Commission has allowed LHQW for the treatment of COVID-19 [282]. The efficacy of this compound against COVID-19 has been reported in several in vitro and human articles [282]. According to the clinical trial effectiveness on humans, LHQW is able to decrease the cytokine release related to lung damage and the inflammatory cascade [283]. Lianhuaqingwen is active in subjects with mild manifestations of fever, fatigue, and cough, and in subjects with severe lesions on the lungs. Several studies reported that Lianhuaqingwen combined with conventional therapies is able to ameliorate the effectiveness of the treatment (91.5% vs. 82.4%); a decrease in the median symptomatic period (seven against 10 days); a reduced period of fever (two vs. three days), cough (7.0 vs. 10 days), and fatigue (3.0 vs. 6.0 days); and the absence of rattling breathing and wheezing compared to the control. Moreover, an increase in tomographic effectiveness has been reported (83.8% vs. 64.1%), and the CRP index improves with a consequent ameliorating of the clinical healing of subjects affected by pneumonia (78.9% vs. 66.2%). Moreover, no severe adverse events related to conventional medicine were reported in the treatment of subjects with severe pneumonia [282,284]. According to the safety and efficacy parameters, Lianhuaqingwen could be administered for the treatment of COVID-19 in order to improve the clinical healing period [282]. 

#### 3.7.5. Gamma Oryzanol

Gamma oryzanol is a substance extracted from rice bran oil and wheat [285]. Recently, based on its anti-inflammatory, antioxidant, and neuroprotective functions, gamma oryzanol has also been used by Traditional Chinese Medicine as a therapeutic for controlling the cytokine storm in COVID-19 [285,286,287,288]. Studies have also shown that gamma oryzanol improves the clinical course in the treatment of some comorbidities related to obesity and reduces cholesterol levels by limiting its absorption from food and, for this reason, it is mainly proposed to treat cases in which cholesterol is high by intervening in the attenuation of the proinflammatory cytokine cascade [287]. Studies have found that γ-oryzanol increases the number of proteins involved in synapses and their expression between neurons, and it is also involved in neuroprotection and antioxidant activity by activating mitochondrial activity and energy metabolism. These results suggest that γ-oryzanol, a natural compound, is able to maintain and strengthen brain function and act as a support by improving the clinical course in patients affected by COVID-19 [288].

#### 3.7.6. Resveratrol

Resveratrol trans-3,5,4′-trihydroxystilbene is a polyphenol characterized by several different benefit effects. This molecule can be obtained from many different plant species such as polygonum cuspidatum, cranberry, red mulberry, and in higher quantities in red grapes and red wine [289]. Resveratrol is poorly soluble in water and scarcely present in the oral cavity [290,291]. The liver is able to metabolize the resveratrol into glucuronides and sulphates and, at high concentrations, it exerts a toxic effect. Resveratrol bioavailability is determined by the typology of diet and interindividual differences and nutritional habitudes [290]. By combining solid lipid nanoparticles (SLNs) and nanostructured lipid carriers (NLCs), resveratrol was trapped by 70%, increasing its stability for up to two months [290]. In vitro studies produced a delayed and longer release of resveratrol at the level of the gastrointestinal tract. Resveratrol is characterized by anti-inflammatory properties while, in cancer patients, it is able to decrease the effect of free radicals and, in subjects affected by cardiovascular diseases, it is able to inhibit the cell apoptosis induced by the reactive oxygen species ROS, activating the AMP-activated protein kinase (AMPK) in the heart muscle cells (H9c2) [203,292,293,294,295]. Moreover, the resveratrol compounds are able to control obesity, type 2 diabetes, and neurodegenerative diseases [203,292,293,294,295]. In fact, the inhibitory capability of resveratrol against bacteria, fungi, and viruses has been reported by several articles. Lin at al. reported in an in vitro study that resveratrol is able to contrast the infections induced by multiple pathogens, such as helicobacter pylori, staphilococcus aureus, or toxoplasma gondii [294]. Resveratrol reported antiviral capability against Epstein-Barr virus (EBV), enterovirus 71 (EV71), and herpes simplex virus (HSV), as well as respiratory viral infections of influenza, respiratory syncytial virus (RSV), rhinoviruses, MERS-CoV (Middle East respiratory virus-coronavirus), human metapneumovirus, and severe acute respiratory syndrome coronavirus (SARS-CoV), as it reduces the inflammatory cascade produced by viral infection [296]. 

As there are already in vitro studies performed by Lin and his team attesting to the positive effects of resveratrol in MERS-CoV infection, it could be considered for use as a therapeutic support for SARS-CoV-2. In fact, these studies show that, by administering resveratrol, cell death (apoptosis) caused by MERS-CoV in vitro is decreased after viral infection [296]. The nucleocapsid protein (N) useful for MERS-CoV replication was also in a lower percentage after administration of resveratrol. Resveratrol at a high concentration of 250 Mm has a lower toxicity (since this substance, by increasing the production of liver enzymes, increases liver stress [297]), but it is secondary to that caused by MERS-CoV infection, which is more severe. Evaluating these results, treatment with resveratrol could be considered a proper therapeutic supportive practice against COVID-19, administering it at a high dosage, up to 250 μM or, at a lower concentration, such as 62.5 μM, repeated several times, every 24 h, for the treatment of MERS-CoV-infected cells [293]. The antiviral mechanisms of resveratrol are recognized in the activation of ERK1/2 receptors (extracellular kinases), promoting cell proliferation and improving the SIRT1 indication (sirtuin 1), all factors related to cell survival and the repair of the subsequent genomic sequence damage to the chain [293]. On the other hand, resveratrol can counteract MERS-CoV-induced apoptosis by stimulating the production of FGF-2 (fibroblast growth factors). Furthermore, MERS-CoV infection can lead to the production of inflammatory cytokines, while resveratrol can reduce inflammation by interfering and inhibiting the NF-KB pathway (nuclear factor kappa-light-chain-enhancer of activated B cells) protein complex functioning as a transcription factor that has a key role in regulating the immune response to infections, inflammatory processes, autoimmune diseases, septic shocks, viral infections, diseases of the immune system, and tumor processes. It has been documented that resveratrol reduced levels of enzymatic cleavage of caspase 3 in patients with MERS-CoV infection [296,298]. This may be the result of direct inhibition of caspase 3 cleavage through restoration of the surviving cell. According to the results of this research, such as the anti-MERS-CoV effect, this effect can also be evaluated in SARS-CoV-2 infection [298]. Its main antiviral mechanisms are based on the inhibition of viral protein synthesis and the inhibition of different modes of transcription and transmission of signals [299]. After these mechanisms, resveratrol determines a “down-regulation” of the apoptosis of the cell induced by MERS-CoV in vitro. Its ability to deactivate the renin–angiotensin system (RAS) in vitro, to reduce ACE1 and AT1R levels and increase ACE2, AT2R and MAS1 levels was found; it also increases cytotoxic T lymphocytes (CTL) and natural killer (NK) immune cells [296,298,300]. All of these findings should be re-evaluated with further studies on the SARS-CoV-2 virus. Since the anti-inflammatory, immunostimulating, and antioxidant properties of polyphenols are recognized, the effects of resveratrol on severe pneumonia caused by SARS-CoV-2 must be evaluated. It has demonstrated clear anti-inflammatory activity and a reduction in the binding of the SARS-CoV-2 coronavirus on the sites of the oral mucosa by resveratrol, both of which could be evaluated further [289,299,300,301,302].

A group of researchers from the Department of Pharmacy of the Federico II University of Naples studied the antioxidant properties of a mixture of polyphenols, rich in resveratrol, present in the pomace of Aglianico grapes extracted with particular methods and microencapsulated to allow greater absorption in the intestine.

The protocol to be submitted to AIFA provides for the use of taurisol in people with COVID-19 in the form of an aerosol. This protocol produced a reduction in inteleukin-6, reducing the inflammatory state. This should avoid the occurrence of an acute respiratory crisis that requires forced ventilation in the ICU, and allow sufficient lung oxygenation, which can allow patients to have time to produce their own antibodies necessary to block the viral infection and allow healing with a pauci or asymptomatic clinical course [303].

## 4. Discussion

The continuing uncertainty about the course of the pandemic and whether or not a third wave of infection will erupt in the near future has pushed extraordinary efforts and new initiatives. Laboratories and researchers around the world are working hard to find solutions to treat and prevent COVID-19, and efforts have been made towards possible solutions for, first, investigating the use of existing drugs and, second, searching for novel therapies able to contrast the disease effectively and efficiently. Thus, there is the need to create a central strategy not only nationally but at global level which convoys the financial funds required to direct and coordinate innovations for worldwide implementation [304]. 

Some innovations have been proposed for trying to prevent new transmissions and contagiousness. Energies have been put together to create modern tools and medical devices that allow for tracing and catching the virus at a very early stage. Many countries such as China, Korea, and Japan are using telemedicine and AI-assisted CT screening tools to help identify patients with pneumonia due to COVID-19. There are procedures still under exploration which use electronic or optical readouts for early detection of the virus in any samples within minutes, and there are sophisticated micro- and nanofluidic systems which accelerate diagnosis with high precision that can be used in hospital wards, at home, or from family medical doctor’s cabinet [304,305]. 

Existing initiatives for COVID-19 diagnostics, vaccines, and therapeutics are “de facto” encouraging an opening up of hope for the near future. Currently, COVID-19 therapies are generally based on two categories: the antivirals that directly target the virus and immune modulators which help the immune system to fight the virus or persuade the immune system from overreacting dangerously [304]. This narrative review provides an overall comprehensive outline of the evidence for the treatments of COVID-19 up to November 2020, together with a broad list of drug and trials. As per the results, the certainty of the evidence was promising but low. Antivirals, general corticoids, Hydroxycloroquine, Heparine, immune modulators (Tocilizumab, Baricitinib), plasma, and stem cells (both autograft and allograft) probably reduce the risk of death, mechanical ventilation, and duration of hospitalization. The overall results guided almost to a complete recovery and discharge from hospital. Therefore, a kind of reasonable certainty exists that makes those procedures valid, or at least of some help. Sheahan et al. reported, in cells culture and in vivo on mice, the effectiveness of antiretroviral drug and interferon-beta administration against MERS-CoV, while the authors reported that Remdesivir and interferon beta increased antiviral activity; RDV increases pulmonary activity and decreases lung viral loads and severe pathology [62]. 

Remdesivir, for instance, reduces both the time to symptom resolution and the duration of mechanical ventilation, but it remains uncertain whether it has any effective impacts on mortality and different outcomes which are important to patients. In addition, a few side-effects were noted for heart electrical conductivity. It is also necessary to stress that the majority of Remdesivir treatment data were obtained from randomized controlled trials sponsored by a pharmaceutical company, while evidence from randomized controlled trials in patients with COVID-19 has so far given low definitive confirmation about its adverse effects [306]. The use of a repurposed drug in combination with other approaches has been proposed to perform a protocol which is able to contrast both the virus’ virulence and the host response involved in determining COVID-19 severity. In this way, the validation of these pharmacological approaches needs more studies and RTCs in the absence of specific antiviral drugs with a low toxicity, high compliance, good tolerance, and efficacy against SARS-CoV-2 infection which are approved for clinical use. Similarly, randomized trials that compared Hydroxychloroquine with standard care and placebo revealed that the initial final data suggested a low positive impact on reducing mortality risk, mechanical ventilation, or duration of hospitalization. However, individuals who received Hydroxychloroquine showed a symptom resolution time shorter than patients who received standard care. The weak point of these studies was uniquely related to the size of the trial samples, which were too small to be considered statistically significant. In addition, data did not show the degree of the adverse effects [50,306].

Doubts and worries of new options are thus based on the scarcity of solid data from randomized controlled trials, which are the “gold standard” in science to prove whether a therapy is safe and effective. Overall, the obtained results from trials have not been convincing, due to the relatively small numbers supported by low quality evidence, according to scientists and health professionals [50,307].

The overall lack of significant outcomes may also be a consequence of poor strategic and scalable strategies that target areas of greatest need, supported by an inadequate central coordination with command in public health surveillance. It should be stressed that there is inadequate financial support to sustain an international coordinated strategy for COVID-19 therapeutic advances and innovations. Therefore, the need for a systematic forecasting system at an international level for predicting future disease outbreaks and pandemics has become the greatest priority.

Present challenges open the debate for a synchronized execution of worldwide innovations in which the coordination between international authorities, state, and local public health departments is essential in order to lead an improvement in the delivery of contact tracing programs [307]. Certainly, for an early and safe diagnosis in the immediate identification of cases affected by SARS-CoV-2, and a differential diagnosis between SARS-CoV-2 and the less aggressive form of the HCoV virus of the same group of Coronaviridae family members, it would certainly be useful and fundamental during this pandemic to have a reliable and rapid test available, while also considering that, in the winter months, the HCoV virus is widespread all over the world. Pham VH, Gargiulo Isacco C, Nguyen KCD et al. have published and registered a patent in Europe, USA, China, and Honk Kong, which is a new test called: “Rapid and sensitive diagnostic procedure for multiple detection of pandemic Coronaviridae family members SARS-CoV-2, SARS-CoV, MERS-CoV and HCoV: a translational research and cooperation between the Phan Chau Trinh University in Vietnam and University of Bari ‘Aldo Moro’ in Italy” [307]. This invention relates, from a more general point of view to diagnostic methods and devices intended for the detection of viruses of the Coronaviridae family. In cases of epidemics or pandemics such as those caused by viruses of the Coronaviridae family (also called coronavirus), such as SARS-CoV-2, SARS-CoV, MERS-CoV, and HCoV, it is important to be able to establish the health conditions of potential suspected cases quickly and effectively, not only in order to save lives but also to have knowledge of the spread of the infection and take the necessary health measures. The idea behind the invention is possible thanks to the aid of the rRT-PCR test for the quantitative detection of the nucleic acid of a plurality of viruses representative of some pathologies related to the Coronaviridae family, in respiratory samples of the upper and lower tract (such as nasopharyngeal or oropharyngeal swabs), sputum, lower respiratory tract aspirate, bronchial alveolar lavage, and nasopharyngeal lavage/aspirate or nasal aspirate, collected from individuals suspected of having COVID-19 by their healthcare provider [307].

The advantages of this test are as follows: (1) An enzymatic stabilizer which allows for the pre-mixing of everything is used in the reaction and, therefore, the operator does not need to prepare the mix for PCR; (2) the selected primers are targeted to the short fragment, and this can allow the operator to reduce the time for the PCR step to less than 1 h; (3) the amplification cycle of the RNA targets of the different coronaviruses occurs in a “One-Step” amplification procedure via MPL1 and MPL2. In this single process, both false negatives and false positives are highly identified. The applications of this test are as follows: The rRT-PCR method confirms the full identification of the virus itself, allowing the exclusion of the presence of contaminations or false positives (exclusion of virus fragments in host endothelial cells) or false negatives (exclusion of fragments of the virus that cannot be identified because identifiable copies). The current “pandemic” scenario (COVID-19) requires robust and reliable diagnostic tests in order to proceed with the necessary decision-making process. A reliable and sensitive test such as that of the present invention will facilitate the organization and definition of all countermeasures to deal with an epidemic or pandemic and its consequences [307].

### Future Orientation for Primary Prevention

The SARS-CoV-2 is able to bind its spike protein (S) to transmembrane angiotensin I, converting the activated enzyme 2 (ACE2) from the transmembrane serum protease 2 (TMPRSS2). The proteins ACE2 and TMPRSS2 are located on the cell membrane in cholesterol-rich lipid rafts [308]. There are two main mechanisms involved in SARS-CoV-2 pathogenesis: entry into cells through endocytosis and the initiation of an exaggerated inflammatory response (cytokine storm) [309]. For this reason, intervening in the mechanism of endocytosis has become an important objective in combating SARS-CoV-2 [310]. Some studies have shown that substances of natural origin, such as cyclodextrins, make many types of viruses less infectious, including the coronavirus family, by interfering with the virus-mediated lipid rafting endocytosis on the membranes of human host cells. Alpha-cyclodextrins preferably bind to saturated fatty acids which, together with cholesterol, form lipid rafts. However, they are unable to eliminate cholesterol. Therefore, it is thought that alpha cyclodextrins can modify, in a targeted manner, the structure of lipid rafts [311]. By evaluating these invasive characteristics of the virus, a spray was produced containing food supplements, compounds that would inhibit both endocytosis and the inflammatory response, helping to reduce the viral load in the oral cavity. The Endovir stop spray, approved by the Italian Ministry of Health, contains α-cyclodextrin, due to its ability to sequester sphingolipids which, together with cholesterol, form the lipid rafts where ACE2 is located. There is also Hydroxystyrosol, extracted from olive leaves and fruit, with anti-inflammatory and anti-oxidant properties [312]. Hydroxytyrosol (HT), a small molecule phenolic compound, has been shown to act against influenza viruses, the Newcastle disease virus, and HIV1. Hydroxytyrosol interacts with the plasma membrane, localizing itself at the level of the hydrophilic heads. This would confer protection against oxidative stress and modify the chemical–physical properties of the membrane [313]. The mechanism of the antiviral effect of HT suggested that the presence of a viral envelope was necessary. The bioactive component of the olive extract, Hydroxytyrosol, shows a moderate average binding affinity for SARS-CoV-2. Hydroxytyrosol has an interaction with the cell membrane and is involved in the regulation of the endocytosis process [314]. The MTT test revealed that the spray is not cytotoxic. The ORAC test showed the spray’s antioxidant capacity. A study was carried out on 87 volunteer individuals with a negative test for COVID-19 and with different clinical characteristics and different drugs taken. None of these individuals experienced any side effects or symptoms of the disease after using the spray for a week [315]. Fifty healthy volunteers at the highest risk of SARS-CoV-2 infection from Northern Cyprus and six SARS-CoV-2 positive individuals were enrolled in another study. The 50 volunteers after 15 days did not test positive for SARS-CoV-2 after administering the compound for two weeks, despite being at a higher risk of infection than the general population. In the cohort of six positive patients, two patients were given the spray and tested negative after five days, and untreated patients turned negative after ten days [316]. The protocol provides for the use of four sprays per day (0.5 mL) as prophylaxis: one in each nostril and two in the oral cavity, capable of administering approximately 0.125 mL ± 10% for each spray, for a quantity of 0.375 mg of CD (±10%) and 1.25 mg of HT (±10%) per spray, 6 h apart, avoiding introducing food immediately afterwards. However, distancing, masks, disinfectant gel, and vaccines remain the priority devices. In Prague, at the European Biotechnology Congress 2020, the European Biotechnology Thematic Network Association the ENDOVIR STOP was awarded as the best anti-COVID-19 product of the year 2020 [317]. 

## 5. Conclusions

In conclusion, the COVID-19 pandemic provoked an unprecedented crisis for public health and the world economy. It represents the largest global public health crisis of this century and it has had a huge impact all over the word. The high quantity of affected subjects that contracted the virus and became symptomatic after a relative short period has compromised healthcare facilities, which responded to the pandemic by reorganizing hospitals and engaging doctors from different specializations to tackle the emergency. The efficacy of different drug class combinations has produced an amelioration of the clinical course of COVID-19 patients, as well as a decrease in symptom severity and overall mortality. More randomized clinical trials are necessary to identify novel pharmacospecific therapies and infection prevention protocols, and to improve their efficacies and safety for clinical application.

## Figures and Tables

**Table 1:** Summary of the Boolean keyword search conducted on electronic databases.

	Electronic Search Keywords and Indicators
Keywords:	Advanced keyword search: ((“COVID-19” OR “2019-nCoV” OR “coronavirus” OR “SARS-CoV-2”) AND drugs) AND (Therapy/Narrow[filter])
Databases	PubMed (Medline), EMBASE, Google Scholar, UpToDate, and Web of Science

**Table 2:** Summary of the in vivo studies included about antiviral drug therapies for viral infections.

Antiviral Drugs				
Authors	Drug	Study Design	Administration Protocol	Results
Chan et al. [51]	Lopinavir, Ritonavir	Cohort study	Lopinavir 400 mg/Ritonavir 100 mg oral administration twice a day.	Decrease in the death rate 2.3% and 0% intubation rate.
Chu et al. [52]	Lopinavir, Ritonavir	Cohort study	Lopinavir 400 mg/Ritonavir 100 mg oral administration twice a day for 2 weeks.	Mild adverse reactions, favorable clinical response of Lopinavir/Ritonavir therapy.
Lim et al. [53]	Lopinavir, Ritonavir	Clinical report	Lopinavir 200 mg/Ritonavir 50 mg oral administration twice a day for 2 weeks.	Lopinavir/Ritonavir is recommended for high-risk population groups for COVID-19.
Young et al. [54]	Lopinavir, Ritonavir	Clinical report	Lopinavir 400 mg/Ritonavir 100 mg oral administration twice a day for 2 weeks.	No subjects with a severe respiratory pathology/1 subject supplemental oxygen administration.
Cao et al. [55]	Lopinavir, Ritonavir	Randomized clinical trial	Lopinavir 400 mg/Ritonavir 100 mg oral administration.	Mortality rate similar between the Lopinavir–Ritonavir protocol and the control group, but shorter time of clinical improvement.
Furuta et al. [56]	Favipiravir	In vivo animal study	Oral administration 100 mg/kg four times a day.	Favipiravir useful and selective against influenza virus infections.
Furuta et al. [57]	Favipiravir	In vivo animal study	Oral administration 30 mg/kg/twice a day and four times a day.	Anti-viral activities against influenza H3N2, H3N2 or H5N1.
Sissoko et al. [58]	Favipiravir	non-randomized clinical trial	Oral administration loading dose: 6000 mg; dose: 2400 mg/d for 9 days.	Mean decrease in Ebola viral load of 0.33 log10 copies/mL/day.
Chinello et al. [59]	Favipiravir	Case report	Oral administration loading dose: 6000 mg; dose: 1.200 mg/d for 9 days.	Mean decrease in Ebola viral load of 0.33 log10 copies/mL/day.
Kumagai et al. [60]	Favipiravir	QT study	Single oral doses 1200 and 2400 mg.	No detectable effects on the QT/QTc interval.

**Table 3:** Summary of the in vivo studies included about repurposed antimalarial drug therapies for viral infections.

Repurposed Drugs				
Authors	Drug	Study Design	Administration Protocol	Results
Chen et al. [89]	Hydroxychloroquine	Randomized clinical trial	Dose: Hydroxychloroquine 400 mg/d for 5 days oral administration	Shorter time to clinical recovery and promotion the absorption of pneumonia.
Gautret et al. [90]	Hydroxychloroquine, Azithromycin	Non-randomized clinical trial	Dose: Hydroxychloroquine 600mg/d azithromycin 250 mg/d oral administration	Azithromycin/Hydroxychloroquine protocol was more efficient for virus elimination.
Momekov et al. [81]	Ivermectin	Pharmacokinetic study	Dose: 150–800 µg/kg up to 2000 µg/kg oral administration	Concentrations not attainable against SARS-CoV-2

**Table 4:** Summary of the in vivo studies included about convalescent plasma treatments for viral infections.

Convalescent Plasma				
Authors	Drug	Study Design	Administration Protocol	Results
Hung et al. [113]	Convalescent Plasma	Prospective cohort study	Convalescent plasma-neutralizing antibody titer ≥ 1:160	Reduced respiratory tract viral load, cytokine response; additionally, mortality against H1N1
Kraft et al. [115]	Convalescent Plasma, TKM-100802	Clinical reports	TKM-100802 infusion 0.3 mg/kg/convalescent plasma	No serious long-term sequelae against Ebola virus disease
van Griensven et al. [116]	Convalescent Plasma	Non-randomized clinical trial	Convalescent plasma 500 mL, unknown neutralizing antibodies titer	Higher cycle-threshold values, a shorter duration of symptoms of Ebola virus disease
Duan et al. [117]	Convalescent Plasma	Clinical reports	Convalescent plasma 200 mL-neutralizing antibody titers 1:640	Decreased symptoms and increase in oxyhemoglobin saturation within 3 days in severe COVID-19 subjects

**Table 5:** Summary of the in vivo studies included about immunomodulator treatments for viral infections.

Immunomodulators				
Authors	Drug	Study Design	Administration Protocol	Results
Xu et al. [128]	Tocilizumab	Clinical trial	Lopinavir/Ritonavir 200/50 mg twice a day, oral administration; IFN-α (5 million unit/2 mL aerosol twice a day; tocilizumab dose load: 4–8 mg/kg to 400 mg intravenous administration	Improved the clinical outcome in severe cases of COVID-19, reduced mortality rate

**Table 6:** Summary of the in vivo studies included about corticosteroid treatments for viral infections.

Corticosteroids				
Authors	Drug	Study Design	Administration Protocol	Results
Arabi et al. [160]	Corticosteroids; Hydrocortisone-equivalent doses (methylprednisolone, 1:5; dexamethasone, 1:25; prednisolone, 1:4)	Multicenter clinical trial	Corticosteroid therapy initiation	No difference of 90-day mortality rate if associated with delayed MERS-CoV RNA clearance
RECOVERY Collaborative Group [161]	Dexamethasone Methylprednisolone Prednisone	Randomized clinical trial	Dose of 6 mg (eq. 160 mg hydrocortisone, 32 mg Methylprednisolone, 40 mg Prednisone) EV daily up for 10 days	The drug protocol produced a decrease in 28-day mortality in severe intensive therapy patients

**Table 7:** Summary of the in vivo studies included in anticoagulants treatments for viral infections.

Anticoagulants				
Authors	Drug	Study Design	Administration Protocol	Results
Tang et al. [167]	Low molecular weight heparin	Clinical trial	Low molecular weight heparin for at least 7 days	Better prognosis in severe COVID-19 patients

**Table 8:** Summary of the in vivo studies included about stem cells treatments for viral infections.

Stem Cells Treatment				
Authors	Drug	Study Design	Administration Protocol	Results
Gargiulo et al. [175]	Autologous peripheral blood stem cells	Case report	Anti-retroviral therapy plus stem cell therapy with 0.5 mL of human placenta for 4 and 1/2 months of treatment	Decreased HIV viral activity with a total recovery of pneumonia and skin infection.
Xu et al. [176]	Menstrual blood-derived MSCs	Clinical trial	Allogeneic, menstrual 9 × 10^7^ blood-derived MSC therapy, and concomitant therapies at 0, 3, and 5 days	A significant decrease in dyspnea as early response. No difference in adverse events (AEs) between test and control group.
Shi et al. [177]	Umbilical cord-mesenchymal stem cells (UC-MSCs)	Randomized clinical trial	4 × 10^7^ UC-MSCs per infusion and concomitant therapies on days 0, 3, and 6	Decrease in lung lesion and solid component lesion volume after the treatment. Similar adverse events ratio compared to the placebo.
Lanzoni et al. [178]	Umbilical cord-mesenchymal stem cells (UC-MSCs)	Randomized clinical trial	100 ± 20 × 10^6^ UC-MSCs per infusion at day 0 and day 3 and concomitant therapies	No difference in adverse events. A significant decrease in inflammatory cytokines in the test groups.
Meng et al. [179]	Umbilical cord-mesenchymal stem cells (UC-MSCs)	Randomized clinical trial	3 × 10^7^ UC-MSCs per infusion	UC-MSCs infusion in moderate and severe COVID-19 subjects is safe and well tolerated.

**Table 9:** Summary of the in vivo studies included about adjuvant and antioxidant treatments for viral infections.

Adjuvants and Antioxidants				
Authors	Drug	Study Design	Administration Protocol	Results
Montoya et al. [195]	Low-dose methylphenidate hydrochloride with a mitochondrial modulator	Randomized clinical trial	Methylphenidate hydrochloride oral 5 mg twice a day/1 week-10 mg twice a day/2 weeks; Mitochondrial modulator, oral 4 tablets twice a day	Decrease in fatigue and concentration disturbance related to myalgic encephalomyelitis and chronic fatigue syndrome
Comhaire et al. [196]	Sodium dichloroacetate	Pilot study	Dosage oral one tablet/day for 1 month	Benefit of nutriceutical treatment by sodium dichloroacetate against myalgic encephalopathy/chronic fatigue syndrome symptoms
Carr et al. [197]	High-dose vitamin C	Clinical trial	Oral dosage 24 g/day of IV vitamin C/7 days	A reduction in symptoms in the high-dose treatment. No difference among the prognosis and other clinical outcomes of COVID-19
